# Establishment of One-Pot ERA-CRISPR/Cas12a-Based Rapid Visual Assays and a TaqMan Quantitative PCR Assay for *Lactococcus garvieae*

**DOI:** 10.3390/microorganisms14040830

**Published:** 2026-04-05

**Authors:** Haoyu Wang, Heng Sun, Feiming Chen, Zhiyuan Huang, Yu Chen, Xiaofeng Chen, Dogbey Rejoice Abla, Zhi Zhang, Huajian Lin, Liqun Wang, Yucong Huang

**Affiliations:** 1 College of Fisheries, Guangdong Ocean University, Zhanjiang 524088, China; haoyuwangmail@foxmail.com (H.W.); hengsuen@foxmail.com (H.S.);; 2 Guangdong Provincial Key Laboratory of Aquatic Animal Disease Control and Healthy Culture & Key Laboratory of Control for Diseases of Aquatic Economic Animals of Guangdong Higher Education Institutes, Zhanjiang 524088, China; 3 Guangdong Provincial Animal Disease Prevention and Control Center, Guangzhou 510230, China

**Keywords:** *Lactococcus garvieae*, enzymatic recombinase amplification, CRISPR/Cas12a, lateral flow assay, one-pot assay

## Abstract

*Lactococcus garvieae* is a major bacterial pathogen responsible for lactococcosis outbreaks in aquaculture, resulting in substantial economic losses worldwide. Accurate identification of *L. garvieae* remains challenging because of its genetic similarity to other Lactococcus species and the limited field applicability of many existing molecular diagnostic methods. Therefore, there is an urgent need for a rapid, highly specific, and field-deployable analytical method that enables accurate identification of *L. garvieae* outside conventional laboratory settings. In this study, a one-pot analytical strategy integrating enzymatic recombinase amplification (ERA) with CRISPR/Cas12a detection was developed, enabling fluorescence or lateral flow dipstick (LFD) readouts within a single closed reaction tube. The one-pot ERA-CRISPR/Cas12a assays achieved a detection limit of 10 copies/reaction. When combined with a rapid DNA release protocol, qualitative detection could be completed within 50 min without the need for sophisticated instrumentation. In parallel, a TaqMan quantitative PCR assay was established as an analytical benchmark, exhibiting a detection limit of 20 copies/reaction with high linearity and good reproducibility. Clinical evaluation using 136 diseased fish samples demonstrated full concordance between the one-pot ERA-CRISPR/Cas12a and qPCR assays, with both methods achieving a positive detection rate of 23.5% (32/136). In addition, the ERA-CRISPR/Cas12a platform was successfully validated under simulated field conditions using a portable reaction device. This study presents a rapid and field-deployable CRISPR-based platform for the early detection and epidemiological surveillance of lactococcosis.

## 1. Introduction

*Lactococcus garvieae*, a Gram-positive opportunistic pathogen within the family Streptococcaceae, along with closely related species such as *Lactococcus petauri* and *Lactococcus formosensis* [1,2], is recognized as a primary causative agent of lactococcosis in global aquaculture [3]. This pathogen has been documented to infect a variety of economically important freshwater and marine fish species, including *Rachycentron canadum* [4], *Trachinotus* spp. [5,6], * Oreochromis niloticus* [7,8], *Oncorhynchus mykiss* [9,10,11], and *Seriola dumerili* [12]. Under intensive farming conditions, infection can lead to mortality rates exceeding 50% [13,14], resulting in substantial economic losses.

Clinically, fish infected with *L. garvieae* are characterized by hemorrhagic lesions in the eyes, maxillofacial regions, and fin bases. As the infection progresses to advanced stages, it often leads to systemic hemorrhage, visceral petechiae, and abdominal fluid accumulation [15]. These clinical manifestations closely resemble those observed in common streptococcal infection of fish, thereby posing significant challenges for differential diagnosis. Beyond its impact on aquaculture, *L. garvieae* has been identified as a potential zoonotic agent [16,17,18], with documented cases of infective endocarditis in humans associated with handling or consumption of raw fish contaminated with this pathogen [19,20]. Notably, several susceptible fish species, including *O. mykiss*, *S. dumerili*, and *R. canadum* are commonly consumed raw as sashimi, which raises additional concerns regarding food safety and potential zoonotic transmission risk and underscores the importance of accurate and timely detection.

Conventional diagnostic approaches for *L. garvieae* typically include routine microbial isolation, biochemical testing, serological assays, polymerase chain reaction (PCR) [21] and quantitative PCR (qPCR) [22]. However, these methods are often time-consuming and require specialized laboratory equipment. However, recent studies have indicated that traditional diagnostic tools, including commercial biochemical systems, MALDI-TOF, and molecular assays targeting conserved regions such as the 16S rRNA gene or the 16S-23S rRNA intergenic spacer, lack sufficient specificity to reliably distinguish *L. garvieae* from closely related species [23,24]. Although TaqMan-based multiplex qPCR assays capable of differentiating *L. garvieae* have been reported [24,25], such methods remain dependent on expensive instrumentation and well-trained technicians, which limits their applicability in routine aquaculture operations and point-of-care (POC) diagnostic settings.

Isothermal amplification technologies have emerged as attractive alternatives for rapid pathogen detection, owing to their fast amplification kinetics, low operational cost, and compatibility with portable platforms [26,27]. By eliminating the requirement for thermal cycling [28,29], these methods are particularly suitable for field diagnostics. Techniques such as recombinase polymerase amplification (RPA), multi-enzyme isothermal rapid amplification (MIRA), recombinase-aided amplification (RAA), and enzymatic recombinase amplification (ERA) have been successfully applied to the detection of aquatic pathogens, including *Vibrio parahaemolyticus* [30], *Streptococcus iniae* [31], and *Enterocytozoon hepatopenaei* [32]. Despite these advantages, the high amplification efficiency also increases susceptibility to aerosol contamination and the risk of false-positive results [33]. Achieving an appropriate balance between sensitivity and operational robustness remains a significant challenge for POC applications.

CRISPR/Cas-based detection platforms have recently emerged as powerful tools for pathogen diagnostics due to their high specificity and programmable targeting capabilities. Representative CRISPR-Dx diagnostic platforms such as SHERLOCK v2 [34] and DETECTR [35], have demonstrated enhanced sensitivity and specificity by coupling target pre-amplification with CRISPR/Cas systems. In the DETECTR platform, recognition and cleavage of the target sequence by the Cas12a–crRNA complex activate trans-cleavage activity, resulting in the indiscriminate cleavage of non-specific ssDNA reporters [35], enabling robust signal amplification [36]. In these platforms, crRNAs are designed to target amplified regions and typically tolerate 1–2 bp mismatches [37], providing dual recognition at both the amplification and CRISPR-mediated detection stages. This dual-layer specificity enhances diagnostic accuracy and reduces false-positive results compared with conventional isothermal amplification methods alone. Nevertheless, CRISPR/Cas systems without upstream amplification generally lack sufficient sensitivity for detecting low-abundance targets [38,39], necessitating integration with a pre-amplification step.

**Figure 1:**
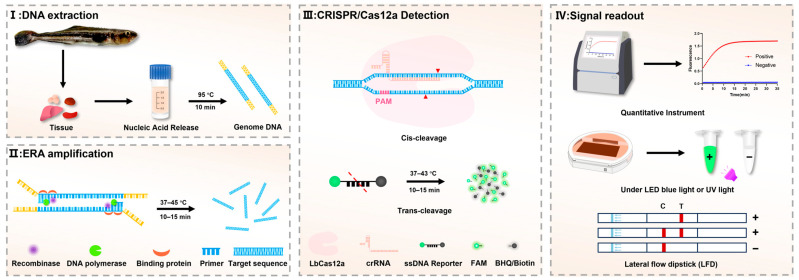
Schematic illustration of the integrated ERA-CRISPR/Cas12a detection platform. The workflow comprises four sequential steps: (**I**) genomic DNA extraction, (**II**) ERA, (**III**) CRISPR/Cas12a-mediated detection, and (**IV**) signal readout. Fluorescence signals can be quantified using specialized instruments or visually assessed under blue LED light, while lateral flow dipsticks provide an alternative instrument-free visualization method.

In this study, we developed a one-pot ERA-CRISPR/Cas12a diagnostic platform for the detection of *L. garvieae*, integrating ERA with CRISPR/Cas technology to enable both fluorescence and lateral flow dipstick (LFD) readout options (Figure 1). In addition, a novel TaqMan qPCR assay was established for accurate quantification of *L. garvieae*. Taken together, these complementary approaches are designed to provide sensitive, specific, and operationally practical solutions for both field-deployable detection and laboratory confirmation of *L. garvieae* infections.

## 2. Materials and Methods

### 2.1. Clinical Samples

A total of 136 diseased fish specimens were collected, including 36 *Trachinotus* spp., 34 *Rachycentron canadum*, 21 *Plectropomus leopardus*, 15 pearl gentian grouper (♀ *Epinephelus fuscoguttatus* × ♂ *Epinephelus lanceolatus*), 12 *S. dumerili*, 8 *Siganus* spp., 7 *Thamnaconus modestus*, and 3 *Selenotoca multifasciata*. Necropsies were performed under aseptic conditions, and liver, spleen, kidney and brain tissue samples were collected from each specimen for DNA extraction. The clinical fish samples were obtained from commercial inshore and offshore mariculture facilities, individual fish farmers, and aquatic product markets. All samples were voluntarily submitted by aquaculture enterprises or individual farmers for diagnostic analysis, which was conducted free of charge. Detailed information on clinical samples is provided in the Supplementary Materials.

### 2.2. Primer, Probe and crRNA Design

For the screening of diagnostic markers, 34 fish-host *L. garvieae* genomes were downloaded from the GenBank database, alongside 50 randomly selected genomes of *L. petauri* and 50 of *L. formosensis*. First, all genomes were uniformly re-annotated using Prokka [40] to ensure consistency. Panaroo [41] was then employed to conduct a pangenome analysis, identifying specific genetic regions by comparing *L. garvieae* with these closely related species. Through strict screening of the pangenome matrix combined with BLASTn (https://blast.ncbi.nlm.nih.gov/Blast.cgi) comparative analysis, a 924 bp gene unique to *L. garvieae* was identified as a candidate diagnostic target (GenBank accession AP009332.1). Primers for PCR, ERA, and TaqMan qPCR assays were designed using Primer Premier 6.0 (Premier Biosoft, Palo Alto, CA, USA). Specifically, the ERA primers were designed in accordance with the manufacturer’s guidelines, with primer lengths of approximately 29–33 bp and an optimal target amplicon size ranging from 100 to 300 bp. Additionally, the crRNA targeting sequences were positioned adjacent to protospacer-adjacent motifs (PAMs; TTTV or TTV, V for A, G, or C). All primers, crRNA, the ssDNA reporter probe, and the TaqMan probe were synthesized by Sangon Biotech Co., Ltd., Guangzhou, China. The full sequences are provided in Table 1.

### 2.3. Construction of the Recombinant Plasmid Standard

The *L. garvieae* strain ATCC 49156 was cultured in Brain Heart Infusion (BHI) broth (Qingdao Hope Bio-Technology, Qingdao, China) and incubated overnight at 28 °C. Genomic DNA was extracted using the TIANamp Bacteria DNA Kit (TIANGEN, Beijing, China). DNA quality and concentration were assessed using a NanoDrop 2000 spectrophotometer (Thermo Fisher Scientific, Waltham, MA, USA) by measuring the OD_260_/OD_280_ absorbance ratio. The target gene fragment of *L. garvieae* was amplified using a T20D thermal cycler (LongGene, Hangzhou, China). The reaction mixture contained 25 µL Phanta Max Master Mix (Vazyme, Nanjing, China), 2 µL of each forward and reverse primers (10 µM), 2 µL DNA template (220 ng/µL), and ddH_2_O to a final volume of 50 µL. PCR cycling conditions were as follows: initial denaturation at 95 °C for 3 min; followed by 30 cycles of denaturation at 95 °C for 30 s, annealing at 58 °C for 30 s, and extension at 72 °C for 30 s; with a final extension at 72 °C for 5 min. PCR products were separated by electrophoresis on 1.5% agarose-TAE gels stained with ethidium bromide, and the target fragments were purified using the SanPrep Column DNA Extraction Kit (Sangon Biotech, Shanghai, China). A standard plasmid was generated by ligating the purified PCR amplicons into the pCE3 vector using the Ultra-Universal TOPO Cloning Kit (Vazyme, Nanjing, China). The ligation reaction was prepared by mixing 2 µL of the TOPO Cloning Mix with 30 ng of the PCR amplicons and ddH_2_O to a final volume of 10 µL. The mixture was incubated at 37 °C for 5 min, followed by transformation into *Escherichia coli* DH5α competent cells. Plasmid DNA was subsequently extracted using the TIANpure Midi Plasmid Kit (TIANGEN, Beijing, China).

### 2.4. Development of ERA-CRISPR/Cas12a Detection Platform

#### 2.4.1. Preliminary Optimization of ERA and CRISPR/Cas12a Cleavage

To facilitate the rational development of a one-pot ERA-CRISPR/Cas12a detection platform, preliminary optimization was performed using a conventional two-step configuration. This approach enabled the independent evaluation of ERA efficiency and CRISPR/Cas12a cleavage performance, thereby avoiding interference between the two processes during parameter optimization.

ERA optimization was performed using the ERA Basic Kit (KS101, Suzhou GenDx, Suzhou, China) with a systematic evaluation of the reaction temperature and amplification time. Each ERA reaction contained one lyophilized enzyme pellet, 20 μL rehydration buffer, 2 μL of each forward and reverse primers (10 μM), 2 μL template DNA, 2 μL MgOAc (350 mM), and DEPC-treated water (Servicebio, Wuhan, China) to a final volume of 50 μL. ERA reactions were incubated for 5, 10, 15, 20 and 25 min to determine the optimal amplification duration. Based on the optimal incubation time identified, the amplification efficiency was further evaluated at different temperatures. The efficiency of ERA under different conditions was indirectly assessed using a downstream CRISPR/Cas12a fluorescence assay. Briefly, 2 μL of ERA product was mixed with 18 μL of CRISPR reaction mixture containing 1 μL LbCas12a (EDE0005, Editgene, Guangzhou, China), 2 μL reaction buffer, 1 μL crRNA (10 μM), 1 μL ssDNA reporter (10 μM), and DEPC-treated water up to 20 μL. The reaction mixture was incubated in a LightCycler 96 detection system (Roche Diagnostics, Basel, Switzerland), and fluorescence signals were continuously recorded in the FAM channel at 30 s intervals over a 30 min period. To optimize CRISPR/Cas12a cleavage efficiency, eight LbCas12a to crRNA molar ratios (1:6, 1:5, 1:4, 1:3, 1:2, 1:1, 2:1, and 3:1) were initially evaluated. Following identification of the optimal ratio (1:4), further optimization was performed using gradient concentrations of both LbCas12a and crRNA (from 25 nM:100 nM to 175 nM:700 nM). Given that elevated ssDNA reporter concentrations contribute to increased background fluorescence, ssDNA reporter concentrations ranging from 100 to 600 nM were evaluated to improve signal discrimination. Signal-to-background (S/B) ratios were calculated based on fluorescence intensities obtained from positive and negative controls. Additionally, CRISPR/Cas12a cleavage activity was also evaluated across a reaction temperature gradient (ranging from 37 to 45 °C) to determine the optimal reaction temperature. The optimized parameters obtained from this preliminary two-step configuration were applied to establish the integrated one-pot ERA-CRISPR/Cas12a detection platform.

#### 2.4.2. Establishment of One-Pot ERA-CRISPR/Cas12a Detection Platform

Based on the optimized parameters obtained from the preliminary two-step configuration, a spatially separated one-pot ERA-CRISPR/Cas12a platform was established as the final analytical format. Physical separation of the amplification and CRISPR/Cas detection components within a single closed tube was employed to prevent premature interaction between reagents.

In the one-pot assay, the ERA reaction mixture was prepared at the bottom of the reaction tube and consisted of 10 μL ERA rehydration buffer, forward and reverse primers at a final concentration of 600 nM each, 1 μL template DNA, 14 mM MgOAc, and DEPC-treated water to achieve a final volume of 25 μL. The CRISPR/Cas12a detection mixture (5 μL), containing reaction buffer, LbCas12a (750 nM), crRNA (3 μM), and ssDNA reporter (3 μM), DEPC-treated water, was dispensed onto the inner surface of the tube cap to maintain spatial separation during the amplification phase. The assembled reaction was incubated at 43 °C for 15 min to allow independent progression of ERA. Subsequently, brief centrifugation was performed to merge the ERA and CRISPR/Cas12a components. Fluorescence signals were continuously monitored at 43 °C for a duration of 30 min.

To facilitate instrument-free readout and field deployment, a portable LFD format was further developed. In this LFD readout, the 3′ BHQ1 quencher on the QF ssDNA reporter was replaced with a biotin modification. Upon completion of the one-pot reaction, the reaction mixture was diluted to 100 μL with ddH_2_O. The diluted sample was loaded onto a commercial lateral flow dipstick (TS104, Suzhou GenDx, Suzhou, China) by immersing the absorbent pad. Results were visually interpreted after 5 min. According to the manufacturer’s instructions, a valid positive result was defined by the presence of both control line (C line) and test line (T line) signals, or by the T line signal alone, conversely, a negative result was indicated by a distinct C line signal in the absence of a T line signal.

#### 2.4.3. Sensitivity and Specificity Analysis of ERA-CRISPR/Cas12a Detection Platform

The concentration of purified recombinant plasmid DNA was quantified using the Qubit™ dsDNA HS Assay kit (Thermo Fisher Scientific, Waltham, MA, USA) according to the manufacturer’s instructions. To evaluate analytical sensitivity, a series of 10-fold serial dilutions of the standard plasmid were prepared in DNA diluent (Sangon Biotech, Shanghai, China), covering a concentration range from 2 × 10^9^ to 2 × 10^0^ copies/μL.

Assay specificity was assessed using genomic DNA extracted from bacterial species associated with aquatic animal diseases. The concentrations of these bacterial DNA templates ranged from 76 to 240 ng/µL, and all specificity tests were independently performed in triplicate. The panel included *L. garvieae* (ATCC 49156 and ATCC 43921), closely related species (*L. petauri*, *L. formosensis* and *Lactococcus lactis*), common aquatic bacteria pathogens (*S. iniae*, *Streptococcus dysgalactiae*, *Streptococcus agalactiae*, *Streptococcus parauberis*, *Mycobacterium marinum*, *Bacillus subtilis*, *Nocardia seriolae*, *Vibrio alginolyticus*, *Vibrio harveyi*, *Vibrio vulnificus*, *V. parahaemolyticus*, *Vibrio campbellii*, *Vibrio anguillarum*, *Photobacterium damselae* subsp. *piscicida*, *Photobacterium damselae* subsp. *damselae*, *Aeromonas hydrophila*, *Aeromonas salmonicida*, *Francisella noatunensis*, *Flavobacterium columnare*, *Edwardsiella piscicida*, *Edwardsiella tarda*, *Shewanella algae*, *Citrobacter freundii* and *Plesiomonas shigelloides*). With the exception of the reference strains, all bacterial isolates were derived from diseased aquatic animals.

### 2.5. Establishment and Optimization of the TaqMan qPCR Assay

#### 2.5.1. Optimal Conditions for the TaqMan qPCR

The TaqMan qPCR assay was optimized using a 20 μL reaction volume consisting of 10 μL Probe Master Mix (Q513-02, Vazyme, Nanjing, China), forward and reverse primers, TaqMan probe, ddH_2_O and DNA template. Amplification was performed on a LightCycler 96 detection system. Following the method described by Sun et al. [42], the annealing temperature, primer concentration, and probe concentration were systematically optimized using plasmid standards maintained at a constant concentration of 1 × 10^4^ copies/μL. The cycling conditions included preincubation at 37 °C for 5 min; initial denaturation at 95 °C for 1 min, followed by 40 cycles of denaturation at 95 °C for 10 s, annealing at 53–61 °C for 30 s. To determine the optimal annealing temperature, amplification efficiency and fluorescence signal intensity were evaluated across the tested temperature range. Subsequently, primer concentrations ranging from 0.2 to 0.8 μM were assessed at a fixed probe concentration of 62.5 nM. Finally, probe concentrations from 62.5 to 500 nM were evaluated under conditions of 59 °C annealing temperature and 0.6 μM primer concentration.

#### 2.5.2. Construction of the Standard Curve for TaqMan qPCR

A standard curve was generated using 10-fold serial dilutions of plasmid DNA ranging from 1 × 10^9^ to 1 × 10^0^ copies/reaction. TaqMan qPCR was performed using these dilutions as templates, and the standard curve was constructed via linear regression analysis of log-transformed copy numbers against their corresponding cycle threshold (Ct) values.

#### 2.5.3. Sensitivity, Specificity, Repeatability and Reproducibility of the TaqMan qPCR Assay

The limit of detection (LOD) was determined under the optimized qPCR conditions using the serially diluted plasmids described above. Specificity was evaluated using genomic DNA extracted from the aquatic pathogens listed in Section 2.4.3, following the optimized protocol. Inter-assay and intra-assay reproducibility were assessed in triplicate across three independent runs using 10-fold serial dilutions of the standard plasmid. The coefficient of variation (CV) was calculated as follows: CV (%) = (standard deviation/mean Ct value) × 100.

### 2.6. Repeatability Evaluation Using L. garvieae Bacterial Suspensions

*L. garvieae* strain ATCC 49156 was cultured in BHI medium at 28 °C until it reached the mid-logarithmic growth phase. Bacterial cell density was estimated by measuring optical density at 600 nm using an Evolution 2000 spectrophotometer (Thermo Fisher Scientific, Waltham, MA, USA). Serial dilutions of the bacterial suspension were prepared in BHI medium. Based on the previously established rapid DNA extraction protocol [43], 100 μL of each diluted bacterial suspension was mixed with 1 mL rapid DNA release reagent (20 mM Tris-HCl, 300 mM NaCl, 1 mM EDTA, 1% SDS, 0.8% PVP-40, thermostable pronase and proteinase K) at a 1:10 ratio and subjected to thermal lysis at 95 °C for 10 min. The resulting lysates supernatants were directly tested using the ERA-CRISPR/Cas12a assays to evaluate assay performance and repeatability under conditions simulating practical diagnostic applications. For comparative analysis, bacterial DNA concentration in the lysates were quantified using the TaqMan qPCR assay developed in this study.

### 2.7. Clinical Application of the One-Pot ERA-CRISPR/Cas12a and the TaqMan qPCR Assay

To further evaluate the clinical applicability of the newly developed detection platforms, a total of 136 diseased fish samples were collected and analyzed. For each specimen, a 100 μL aliquot of homogenized tissue was processed using the rapid DNA release protocol described in Section 2.6. The resulting supernatants were tested using the one-pot ERA-CRISPR/Cas12a-Fluorescence and LFD assays. However, as the rapid sample processing reagent was found to interfere with quantitative performance, it was excluded from template preparation for both the TaqMan qPCR assay developed in this study and the reference TaqMan qPCR assay reported by Shahin et al. (2025) [24]. Instead, nucleic acids intended for qPCR analysis were processed using an automated nucleic acid extraction system (FINDROP, Guangzhou, China) prior to amplification. This experimental design facilitated a direct comparison of qualitative detection outcomes and positive detection rates between the one-pot ERA-CRISPR/Cas12a assays and laboratory-based TaqMan qPCR methods.

### 2.8. Retrospective Detection of Past L. garvieae Isolates from the South China Sea

During epidemiological surveillance of aquaculture sites in the South China Sea between 2020 and 2025, 57 *L. garvieae* isolates were isolated from diseased aquatic animals. Detailed information for each isolate is provided in the Supplementary Materials. To further assess the newly developed detection platforms, all archived isolates were retrospectively tested using the one-pot ERA-CRISPR/Cas12a-Fluorescence assay and the TaqMan qPCR assay established in this study. Additionally, the TaqMan qPCR assay reported by Shahin et al. (2025) [24] was included as an external reference.

### 2.9. On-Site Application

Molecular detection in resource-limited environments is often constrained by the lack of heating equipment and centrifugation facilities. To address this challenge, a portable, integrated detection device compatible with the one-pot workflow was designed and fabricated using a 3D printer (UnionTech, Shanghai, China). The reaction tube consists of two chambers separated by a movable septum, which enables physical isolation of the ERA mixture from the CRISPR/Cas detection reagents during the initial amplification phase. After a 15 min isothermal amplification step, the septum is manually actuated to merge the two chambers, allowing the amplified products to directly activate the CRISPR/Cas12. After an additional incubation period of 5–20 min, results can be obtained either by direct fluorescence visualization under blue light illumination (470–490 nm) with a 510 nm emission filter, or by LFD analysis following the dilution of the reaction mixture. Notably, the entire reaction process can also be completed using body heat alone, such as by holding the device in the hand. This operational flexibility supports decentralized testing and field deployment in aquaculture environments.

### 2.10. Data Statistics and Analysis

All data were presented as mean ± standard deviation. Statistical analyses were performed using GraphPad Prism 10.1 (GraphPad Software Inc., San Diego, CA, USA). 

## 3. Results

### 3.1. Optimization of ERA-CRISPR/Cas12a Detection Platform

**Figure 2:**
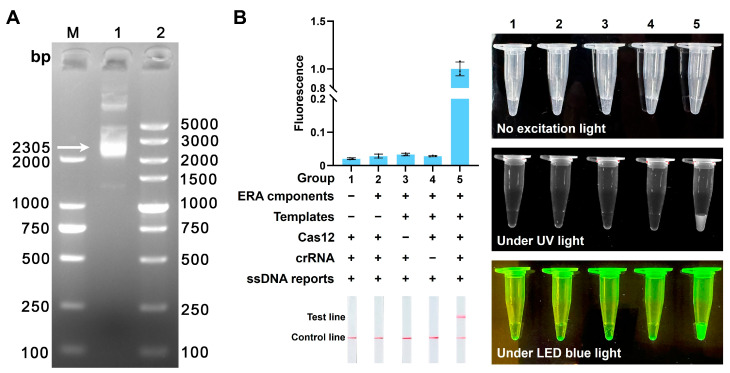
Construction and component validation of the ERA-CRISPR/Cas12a detection platform. (**A**) Agarose gel electrophoresis of the recombinant plasmids. Lane M: DL2000 DNA marker; Lane 1: positive control plasmid; Lane 2: DL5000 DNA marker. (**B**) Component requirement analysis of the ERA-CRISPR/Cas12a detection platform. The plus and minus signs indicate the presence and absence of the corresponding components, respectively.

A recombinant plasmid containing a target sequence was successfully constructed. Its total length of 2305 bp was confirmed by agarose gel electrophoresis (Figure 2A). In subsequent experiments, this plasmid was used as a standard positive control to optimize the detection platform. Component requirement analysis demonstrated that fluorescence and LFD signals were generated exclusively when all essential reaction components were present, confirming the functional integrity and necessity of each component in the ERA-CRISPR/Cas12a detection platform (Figure 2B).

**Figure 3:**
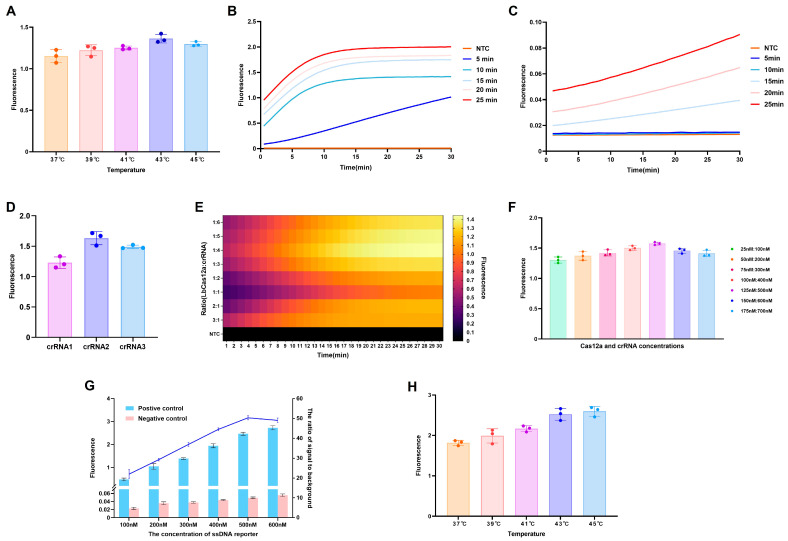
Optimization of reaction conditions for the ERA-CRISPR/Cas12a detection platform. (**A**) Effect of reaction temperature on ERA efficiency. (**B**,**C**) Optimization of ERA amplification time at 43 °C for 5, 10, 15, 20, and 25 min using plasmid templates at 2 × 10^4^ copies/reaction and 2 × 10^0^ copies/reaction, respectively. (**D**) Screening of crRNA candidates based on endpoint fluorescence output. (**E**) Heatmap of fluorescence signals obtained using different LbCas12a to crRNA ratios. (**F**) Determination of optimal LbCas12a and crRNA concentrations. (**G**) Effect of ssDNA reporter concentration on fluorescence intensity and corresponding signal-to-background (S/B) ratios. (**H**) Effect of incubation temperature on CRISPR Cas12a cleavage activity.

To optimize the ERA conditions, the effect of incubation temperature was initially evaluated. ERA reactions conducted at 43 °C generated maximal fluorescence signal in downstream CRISPR/Cas12a assays, indicating optimal amplification performance under these conditions (Figure 3A). Subsequently, the influence of amplification duration was assessed by conducting ERA reactions for 5, 10, 15, 20, and 25 min. When a plasmid template concentration of 2 × 10^4^ copies/reaction was used, strong fluorescence signals were observed across all time points (Figure 3B). However, with a lower template concentration of 2 × 10^0^ copies/reaction, detectable fluorescence signals were consistently obtained only after a minimum amplification duration of 15 min (Figure 3C). Based on these results, to ensure reliable sensitivity for low-abundance targets while maintaining overall assay performance, the ERA pre-amplification condition of 43 °C for 15 min was selected.

Among the crRNA candidates tested, crRNA2 generated the highest fluorescence signal under identical incubation conditions and was therefore selected for subsequent experiments (Figure 3D). Evaluation of various LbCas12a to crRNA ratios revealed that a 1:4 ratio produced the highest fluorescence intensity (Figure 3E). Further optimization through gradient concentrations of both components showed that a combination of 125 nM LbCas12a and 500 nM crRNA yielded the maximal fluorescence intensity (Figure 3F). The effect of ssDNA reporter concentration on assay performance was also evaluated. Although increasing ssDNA reporter concentrations resulted in progressively higher fluorescence intensities, assessment of the S/B value identified 500 nM as the optimal reporter concentration, providing the best discrimination between positive and negative samples (Figure 3G). Optimization of the CRISPR/Cas12a cleavage reaction temperature demonstrated that incubation at 43 °C and 45 °C produced comparable maximum fluorescence signals (Figure 3H). The optimal working conditions for the CRISPR/Cas12a reaction were established as 125 nM LbCas12a, 500 nM crRNA, 500 nM ssDNA reporter, and an incubation temperature of 43 °C.

### 3.2. Evaluation of One-Pot ERA-CRISPR/Cas12a Detection Platform

**Figure 4:**
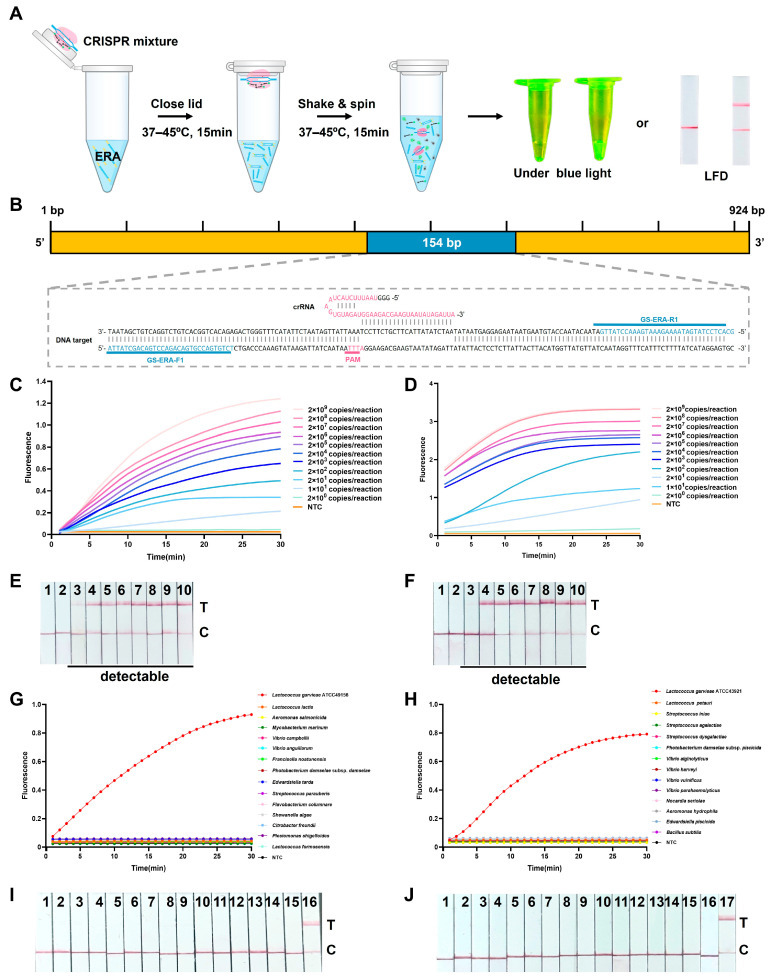
Evaluation of the ERA-CRISPR/Cas12a detection platform. (**A**) Schematic illustration of the one-pot ERA-CRISPR/Cas12a workflow. (**B**) Primer and crRNA Design. The ERA primer binding sites (blue) and the protospacer adjacent motif (PAM, pink) are highlighted in the schematic, illustrating their positions relative to the target sequence. (**C**,**D**) Sensitivity of the one-pot and two-step ERA-CRISPR/Cas12a-Fluorescence assays using serially diluted plasmid DNA. NTC: no template control. (**E**,**F**) Sensitivity of the one-pot and two-step ERA-CRISPR/Cas12a-LFD assays. 1: NTC, 2–10: plasmids containing 2 × 10^0^, 1 × 10^1^, 2 × 10^3^, 2 × 10^4^, 2 × 10^5^, 2 × 10^6^, 2 × 10^7^, 2 × 10^8^ copies/reaction. T: test line; C: control line. (**G**,**H**) Specificity of the ERA-CRISPR/Cas12a-Fluorescence assay. (**I**) Specificity of the ERA-CRISPR/Cas12a-LFD assay. 1–16: NTC, *S. iniae*, *S. dysgalactiae*, *S. agalactiae*, *V. alginolyticus*, *V. harveyi*, *V. vulnificus*, *V. parahaemolyticus*, *N. seriolae*, *P. damselae* subsp. *piscicida*, *A. hydrophila*, *E. piscicida*, *B. subtilis*, *L. petauri* and *L. garvieae* ATCC 43921. (**J**) Specificity of the ERA-CRISPR/Cas12a-LFD assay. 1–17: NTC, *P. shigelloides*, *C. freundii*, *S. algae*, *F. columnare*, *S. parauberis*, *E. tarda*, *P. damselae* subsp. *damselae*, *F. noatunensis*, *V. anguillarum*, *V. campbellii*, *M. marinum*, *A. salmonicida*, *L. lactis*, *L. formosensis*, and *L. garvieae* ATCC 49156.

**Figure 5:**
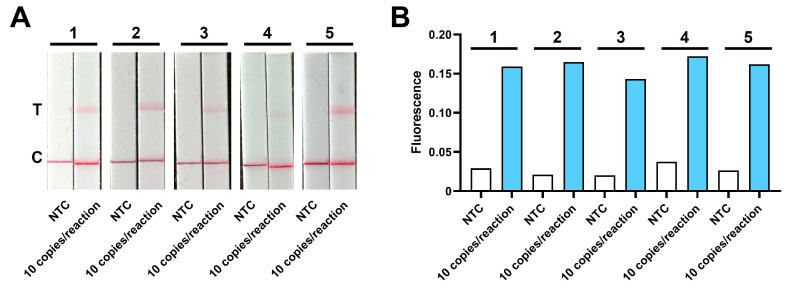
Reproducibility of the one-pot ERA-CRISPR/Cas12a detection platform at the limit of detection. Five independent replicates were analyzed for each method. (**A**) ERA-CRISPR/Cas12a-LFD assay at a template concentration of 1 × 10^1^ copies/reaction. 1–5 represent the five independent replicates. (**B**) ERA-CRISPR/Cas12a-Fluorescence assay at a template concentration of 1 × 10^1^ copies/reaction. 1–5 represent the five independent replicates.

The overall procedure of the spatially separated one-pot ERA-CRISPR/Cas12a detection workflow and the specific gene-targeting region are illustrated in Figure 4A,B. In the fluorescence assays, the two-step ERA-CRISPR/Cas12a assay achieved an LOD of 2 copies/reaction, whereas the one-pot configuration showed an LOD of 10 copies/reaction (Figure 4C,D). Although the one-pot format showed a moderate reduction in sensitivity, both fluorescence assays exhibited comparable performance within the same order of magnitude and provided reliable signal discrimination at low template concentrations. For the LFD assays, identical detection limits of 10 copies/reaction were obtained for both the one-pot and two-step workflows (Figure 4E,F), indicating that workflow integration did not compromise visual detection performance. The target region was analyzed by BLASTn analysis against the NCBI database, which confirmed exclusive sequence homology with *L. garvieae* (GenBank database alignment date: 24 February 2026). Assay specificity was further evaluated using genomic DNA from common aquatic pathogens. The one-pot ERA-CRISPR/Cas12a assays exhibited high specificity, generating distinct fluorescence signals (Figure 4G,H) and positive test lines on the LFD (Figure 4I,J) only in samples containing *L. garvieae*. Non-target pathogens and no template controls produced negligible fluorescence signals and displayed only control line on the LFD. These results confirm the high specificity of the assay for *L. garvieae*, with no cross-reactivity observed with other bacterial species. Given that the one-pot format was established as the final analytical strategy, five independent replicates of the plasmid template (10 copies/reaction) were analyzed to further validate its reproducibility. The results confirmed that both the fluorescence and LFD assays can stably and consistently detect the target at this limit of detection (Figure 5).

### 3.3. Development and Validation of the TaqMan qPCR Assay

**Figure 6:**
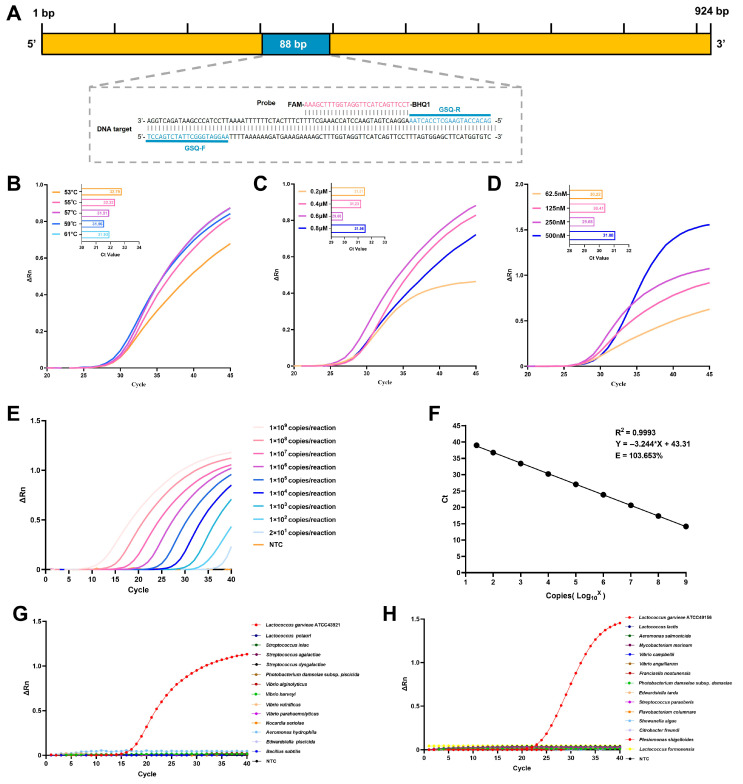
Development and optimization of the TaqMan qPCR assay for *L. garvieae.* (**A**) Primer and probe Design. The TaqMan qPCR primer binding sites (blue) and the probe (pink) are highlighted in the schematic, illustrating their positions relative to the target sequence. (**B**) Annealing temperature was optimized at a primer concentration of 0.6 μM and a probe concentration of 62.5 nM. (**C**) Primer concentration was optimized at an annealing temperature of 59 °C with a probe concentration of 62.5 nM. (**D**) Probe concentration was optimized using the optimal concentrations determined in the previous steps, with a primer concentration of 0.6 μM and annealing temperature of 59 °C. (**E**) Amplification curves generated using 10-fold serial dilutions of plasmid DNA ranging from 1 × 10^9^ to 1 × 10^1^ copies per reaction. (**F**) Standard curve showing the linear relationship between Ct values and the logarithmic plasmid copy number. (**G**,**H**) Specificity assessment of the TaqMan qPCR assay. NTC: no-template controls.

The overall procedure of the TaqMan qPCR detection workflow and the specific gene-targeting region are illustrated in Figure 6A. Key parameters of the TaqMan qPCR were systematically optimized by evaluating annealing temperature, primer concentration, and probe concentration. The optimal annealing temperature was determined to be 59 °C, yielding the lowest Ct values (Figure 6B). A concentration of 0.6 μM for both forward and reverse primers produced the highest amplification efficiency and the lowest Ct value (Figure 6C). Similarly, the optimal probe concentration was identified as 250 nM (Figure 6D). Consequently, the final optimized parameters, namely an annealing temperature of 59 °C, 0.6 μM for both primers, and 250 nM for the probe, were used in all subsequent qPCR analyses. The sensitivity of the optimized TaqMan qPCR assay was evaluated using plasmid standards with concentrations ranging from 1 × 10^9^ to 1 × 10^1^ copies/μL. The LOD of the newly developed TaqMan qPCR for standard plasmid was as low as 20 copies/reaction (Figure 6E). As shown in Figure 6F, the regression equation for the standard curve was Y = −3.244X + 43.31, indicating a strong linear correlation (R^2^ = 0.9993) between the average Ct values and the logarithmic copy number. The calculated amplification efficiency was 103.7%, which complies with the Minimum Information for Publication of Quantitative Real-Time PCR Experiments (MIQE) guidelines [44]. A Ct value of 39.5 was defined as the cutoff threshold for positive detection, whereas signals exceeding this threshold were deemed unreliable due to background noise, ensuring specificity and reliability. Amplification curves were generated exclusively for *L. garvieae*, with no cross-reactivity detected against common aquatic bacterial pathogens or in no-template controls (Figure 6G,H). Analytical precision of the TaqMan qPCR was validated through inter-assay and intra-assay reproducibility assessments, expressed as CVs of Ct values across independent runs. The inter-assay CVs ranged from 0.52 to 1.15%, with average Ct values of 14.73 ± 0.17–39.38 ± 0.28. The intra-assay CVs ranged from 0.22 to 0.56%, with average Ct values of 14.70 ± 0.05–39.02 ± 0.22 (Table 2). These results confirmed high reproducibility and analytical robustness under the optimal cycling conditions.

### 3.4. Repeatability Evaluation of ERA-CRISPR/Cas12a Assays Using L. garvieae Bacterial Suspensions

**Figure 7:**
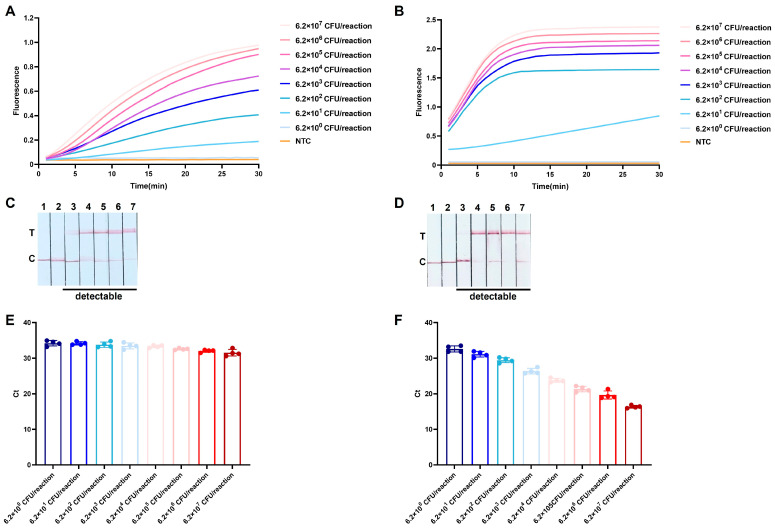
Repeatability and practical performance evaluation of ERA-CRISPR/Cas12a assays using *L. garvieae* bacterial suspensions. (**A**,**B**) One-pot and two-step ERA-CRISPR/Cas12a-Fluorescence assay using bacterial suspensions pretreated with rapid DNA release reagent. NTC: no-template control. (**C**,**D**) One-pot and two-step ERA-CRISPR/Cas12a-LFD assay using bacterial suspensions pretreated with rapid DNA release reagent. 1: NTC; 2–7: gradient bacterial suspension samples containing 6.2 × 10^5^–6.2 ×10^0^ CFU/reaction of *L. garvieae* as templates. T: test line; C: control line. (**E**) TaqMan qPCR quantification results obtained using bacterial suspensions pretreated with rapid DNA release reagent. (**F**) TaqMan qPCR quantification results obtained using bacterial suspensions extracted with the TIANamp Bacteria DNA Kit.

Both the one-pot and two-step ERA-CRISPR/Cas12a-Fluorescence assays exhibited an LOD of 6.2 × 10^1^ CFU/reaction (Figure 7A,B) for *L. garvieae* bacterial suspensions. Consistent detection performance was also observed in the LFD assays, with an identical LOD of 6.2 × 10^1^ CFU/reaction for both workflow configurations (Figure 7C,D), demonstrating comparable sensitivity and repeatability between the integrated and conventional approaches. When the optimized TaqMan qPCR assay was applied to crude lysates prepared using the rapid DNA release reagent, only limited variation in Ct values was observed across the tested bacterial concentrations. The mean Ct values for serial dilutions ranging from 6.2 × 10^7^–6.2 × 10^0^ CFU/reaction were 31.53, 32.00, 32.55, 33.32, 33.44, 33.78, 34.16, and 34.20, respectively (Figure 7E). The lack of a concentration-dependent shift in Ct values indicates that the rapid DNA release reagent is incompatible with accurate quantitative analysis by TaqMan qPCR. In contrast, when DNA extracted from the same bacterial suspensions using a commercial extraction kit was analyzed, a clear correlation between bacterial concentration and Ct values was observed (Figure 7F), confirming the quantitative performance of the qPCR assay under compatible sample preparation conditions.

### 3.5. Examination of Clinical Samples

**Figure 8:**
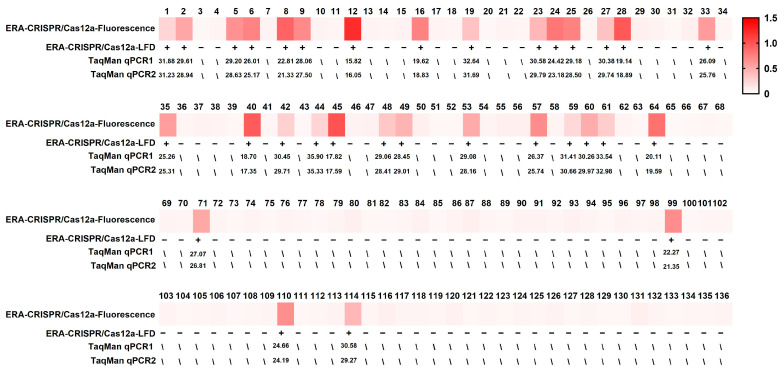
Clinical examination results of fish samples. Specimens analyzed included *Trachinotus* spp. (samples 1–36), *R. canadum* (samples 37–70), *P. leopardus* (samples 71–91), pearl gentian grouper (samples 92–106), *S. dumerili* (samples 107–118), *Siganus* spp. (samples 119–126), *T. modestus* (samples 127–133), *S. multifasciata* (samples 134–136). TaqMan qPCR2 refers to the reference assay reported by Shahin et al. (2025) [24]. TaqMan qPCR1 refers to the assay developed in this study. The plus and minus signs indicate positive and negative results, respectively.

The results of the parallel analysis of 136 clinical diseased fish samples are presented in Figure 8 and Table 3. Using the one-pot ERA-CRISPR/Cas12a platform, both fluorescence and LFD readouts identified 32 positive samples, corresponding to a detection rate of 23.5%. An identical detection rate was obtained with the TaqMan qPCR assay developed in this study, as well as with the reference TaqMan qPCR assay reported by Shahin et al. (2025) [24]. These results demonstrate full qualitative concordance between the rapid detection platform and qPCR assays when applied to naturally infected fish samples, supporting the clinical reliability of the proposed workflow.

### 3.6. Retrospective Detection Results

**Figure 9:**
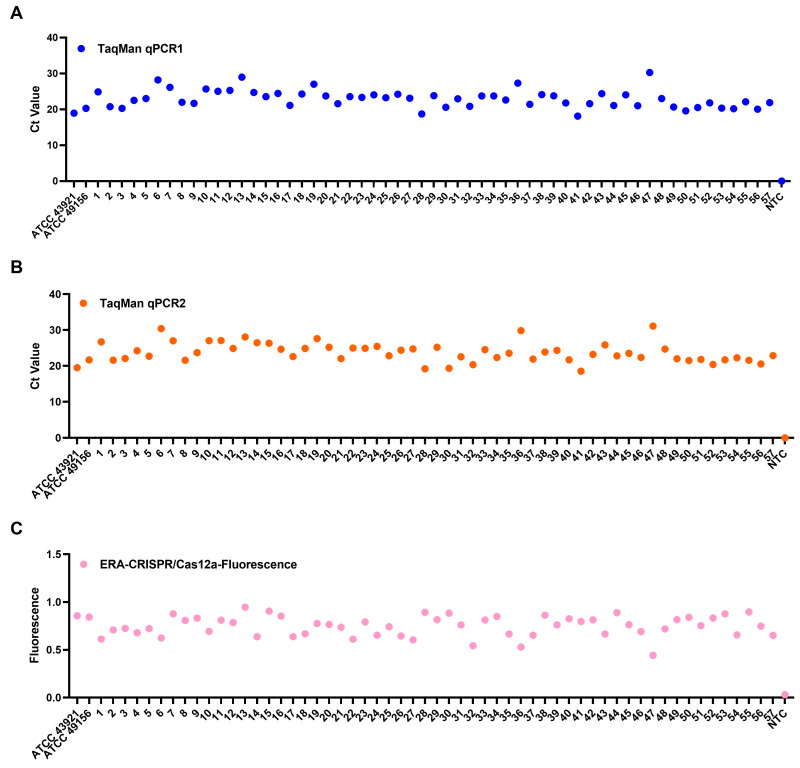
Retrospective of archived *L. garvieae* isolates. (**A**) TaqMan qPCR reported by Shahin et al. (2025) [24]. (**B**) TaqMan qPCR developed in this study. (**C**) One-pot ERA-CRISPR/Cas12a-Fluorescence assay.

All 57 archived *L. garvieae* isolates, collected during epidemiological surveillance, were retrospectively analyzed using the one-pot ERA-CRISPR/Cas12a-Fluorescence assay and the TaqMan qPCR assay developed in this study. For cross-method validation, the TaqMan qPCR assay described by Shahin et al. (2025) [24] was included as an external reference. All isolates were consistently detected as *L. garvieae* by each of the three independent detection methods, with neither amplification signal nor Ct value observed in the negative controls (Figure 9). The complete agreement among the one-pot ERA-CRISPR/Cas12a assay, the newly developed TaqMan qPCR assay, and the reference qPCR method confirms the applicability of newly developed detection platform.

### 3.7. Validation Under Simulated Field Conditions

**Figure 10:**
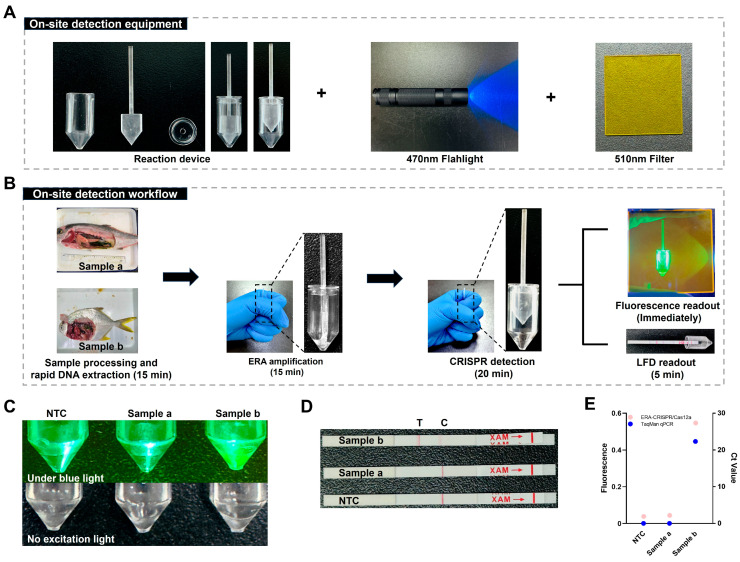
Field application of the one-pot ERA-CRISPR/Cas12a detection platform under simulated on-site conditions. Samples collected from *S. dumerili* (Sample a) and *Trachinotus* spp. (Sample b) were analyzed. (**A**) On-site detection equipment. The reaction tube, movable septum, and lid were fabricated via 3D printing. The upper and lower reaction chambers are physically separated by the movable septum during the amplification phase. Manual actuation of the septum enables chamber merging and initiates the CRISPR/Cas12a detection reaction. (**B**) On-site detection workflow. (**C**) Fluorescence readout of the one-pot ERA-CRISPR/Cas12a assay. (**D**) LFD readout of the one-pot ERA-CRISPR/Cas12a assay. (**E**) Endpoint fluorescence intensity measured using a LightCycler 96 system and TaqMan qPCR detection results.

Two samples collected from *S. dumerili* and *Trachinotus* spp. were provided by individual aquaculture farmers and used for simulated field testing. Following the established field workflow, sample pretreatment using the rapid DNA release reagent and subsequent detection were completed within 50 min using a handheld reaction device, without reliance on external heating or centrifugation equipment (Figure 10A,B). The *Trachinotus* spp. sample yielded a positive result, whereas the *S. dumerili* sample and the negative control produced negative signals (Figure 10C,D). Consistent detection results were obtained using both the ERA-CRISPR/Cas12a-Fluorescence and LFD readouts, demonstrating reliable visual discrimination between positive and negative samples under simulated field conditions. To further validate these on-site results, the reaction mixtures were transferred to centrifuge tubes for endpoint fluorescence measurement using a LightCycler 96 system. The measured fluorescence intensities were in full agreement with the visual readouts, confirming the accuracy of the portable detection workflow. The laboratory qPCR results showed no Ct value for Sample a and a Ct value of 22.3 for Sample b (Figure 10E). Collectively, these results demonstrate the feasibility of a rapid, instrument-free, and visually interpretable assay for on-site detection of *L. garvieae*.

## 4. Discussion

Prior studies have demonstrated that the virulence activation of *L. garvieae* is temperature-dependent, classifying it as a warm-water pathogen [14,15], with infections predominantly occurring under elevated water temperatures. Increasing average water temperatures and prolonged warm seasons have expanded the temporal window for disease outbreaks, posing sustained pressure on aquaculture health management. During epidemiological surveillance conducted in marine aquaculture areas of the South China Sea between 2020 and 2025, a total of 57 bacterial strains associated with lactococcosis were isolated and identified as *L. garvieae*, confirming its status as the predominant etiological agent of lactococcosis in this region and highlighting the urgent need for rapid and reliable diagnostic tools to support early intervention and effective outbreak control and surveillance in aquaculture settings.

From an analytical perspective, accurate identification of *L. garvieae* remains challenging. Existing research demonstrates that conventional 16S rRNA sequencing lacks the sufficient resolution to distinguish *L. garvieae* from closely related species. Moreover, previously reported molecular methods, including PCR [21], qPCR [22] and LAMP [45] targeting the 16S rRNA, ITS region or protein-coding genes, have shown variable specificity [23]. Although *gyrB* gene sequencing is effective for taxonomic identification [1], it is not practical for routine diagnostic applications due to technical and logistical constraints. In this study, a conserved CDS gene identified through comparative genomic analysis was selected as the detection target. Previous studies have suggested that this gene may be associated with the pathogenicity of *L. garvieae* [46]. This region exhibited high species specificity, showed no detectable homology with related lactococcosis pathogens and host genomes, and was consistently present in clinical isolates and reference strains, suggesting that target may serve as a potential species-specific diagnostic marker.

In this study, ERA-CRISPR/Cas12a and TaqMan qPCR assays were established for the specific detection of *L. garvieae*, with no cross-reactivity observed among 26 common aquatic bacterial species. Using plasmid standards, the one-pot ERA-CRISPR/Cas12a-Fluorescence assay achieved an LOD of 10 copies/reaction. This sensitivity was comparable to that reported for MIRA-CRISPR/Cas12a detection of *Lactobacillus acetotolerans* [47] and RPA-CRISPR/Cas12a detection of *Staphylococcus aureus* [48], but slightly lower than RPA-CRISPR/Cas12a for *Candida albicans* (3.8 copies/reaction) [49], while remaining superior to the RT-RPA-CRISPR/Cas12a assay reported for Sugarcane streak mosaic virus (50 copies/reaction) [50]. The one-pot ERA-CRISPR/Cas12a-LFD assay exhibited an LOD of 10 copies/reaction, consistent with the sensitivity reported for RPA-CRISPR/Cas12a-based LFD assays targeting *S. aureus* [48] and *Klebsiella pneumoniae* [51]. In parallel, the newly developed TaqMan qPCR assay showed LOD as low as 20 copies/reaction, which was higher than those reported for *K. pneumoniae* (4.6 copies/reaction) [52] and for *L. garvieae* (9.73 copies/reaction) [24], but lower than those reported for *E. hepatopenaei* (40 copies/reaction) [53], *Aeromonas schubertii* (36 copies/reaction) [54]. These comparisons indicate that the proposed assays achieve sensitivity within the typical range reported for CRISPR-based and TaqMan qPCR diagnostic methods.

Repeatability and practical performance were further evaluated using *L. garvieae* bacterial suspensions pretreated with a rapid DNA release reagent. Both one-pot ERA-CRISPR/Cas12a-Fluorescence and LFD assays consistently detected *L. garvieae* at 6.2 × 10^1^ CFU/reaction. Clinical evaluation of 136 naturally infected fish samples showed complete concordance between ERA-CRISPR/Cas12a assays and both the newly developed and reference TaqMan qPCR assays. These results confirm that ERA-CRISPR/Cas12a platform achieves diagnostic ability comparable to qPCR for qualitative detection, while offering substantially reduced processing time and simplified operation. Consequently, ERA-CRISPR/Cas12a and other CRISPR-Dx are well suited for rapid screening and decentralized surveillance [55,56], whereas qPCR remains the indispensable gold standard for precise quantification, standardization, and epidemiological investigations, serving as a complementary reference method [57].

As summarized in Table 4, most existing molecular diagnostic methods for *L. garvieae* require 1–2 h for analysis, excluding sample preparation, and depend heavily on laboratory infrastructure and trained personnel, which limits their applicability in field and resource-limited settings. Compared with previously reported molecular diagnostic methods for *L. garvieae*, the ERA-CRISPR/Cas12a platform demonstrates competitive sensitivity with markedly improved operational efficiency. Notably, these detection limits were achieved within approximately 35 min, representing a substantial reduction in time relative to PCR and qPCR workflows.

Efficient sample processing remains a critical determinant of the feasibility of field diagnostics [43,49]. The rapid DNA release protocol compatible with ERA-CRISPR/Cas12 assays enables sample preparation without reliance on complex instrumentation. However, residual lytic enzymes (thermostable pronase and proteinase K) in crude lysates adversely have been shown to impair qPCR performance, likely due to thermally stable proteolytic activity interfering with polymerase function or probe hybridization. The development of broadly compatible sample preparation reagents suitable for both isothermal amplification and qPCR remains an important focus for future optimization.

Compared with conventional two-step workflows [62], the one-pot ERA-CRISPR/Cas12 platform established in this study effectively prevents reaction tube reopening, thereby mitigating the risk of contamination. Notably, the one-pot assay exhibits specificity and detection rates comparable to that of qPCR, while offering advantages in operational simplicity, field adaptability and rapid qualitative readouts, thus supporting its application in decentralized pathogen surveillance for aquaculture. While the developed one-pot reaction platform offers significant benefits, several limitations require attention. Primarily, although the closed tube configuration inherently mitigates aerosol contamination risks by removing the need for amplicon transfer, the exceptional sensitivity of the assay necessitates rigorous environmental controls to avoid cross contamination during reagent handling. Furthermore, the expense of CRISPR associated enzymes and isothermal amplification reagents currently exceeds that of conventional PCR and qPCR components, potentially hindering broad implementation in settings with limited resources. Nevertheless, the reliance on simple incubation protocols combined with visual and portable detection methods provides a financially viable alternative by eliminating the dependency on sophisticated laboratory infrastructure Future efforts will focus on integrating CRISPR-based assays into portable and low-cost diagnostic formats, such as microfluidic systems [63] and smartphone-assisted readouts [64], to enable POC diagnostic in resource-limited and field environments [65]. Improvements in reagent stability through strategies such as lyophilization [66] or encapsulation [67] are also expected to enhance assay portability, extend shelf life, and facilitate broader deployment.

## 5. Conclusions

In conclusion, a one-pot ERA-CRISPR/Cas12a platform has been developed for the detection of *L. garvieae*. When combined with a rapid DNA release protocol, this approach enables sample processing and qualitative diagnosis within 50 min, offering a practical solution for rapid on-site detection and pathogen surveillance in various environments. Furthermore, the platform demonstrates potential for adaptation to the detection of other aquatic pathogens. In parallel, a TaqMan qPCR assay has also been established, providing a tool for the diagnosis and epidemiological investigation of lactococcosis. Ultimately, extensive validation of this target and platform across broader clinical cohorts is warranted to fully ascertain their practical reliability among emerging diagnostic alternatives.

## Figures and Tables

**Table 1:** Sequences of the primers, probes, crRNA used in this study.

Assay	Name	Sequence (5′-3′)
PCR	GS-F	TCTCAAAAGAGGGGCGGATT
	GS-R	GCGCTATCCATATCTTTTTAGGGGC
ERA	GS-ERA-F	ATTATCGACAGTCCAGACAGTGCCAGTGTCTCT
	GS-ERA-R	GCACTCCTATGATAAAAGAAATGAAACCTATTG
crRNA	GS-CR1	GGGUAAUUUCUACUAAGUGUAGAUUCAAUAAUUUAGGAAGACGAAGU
	GS-CR2	GGGUAAUUUCUACUAAGUGUAGAUGGAAGACGAAGUAAUAUAGAUUA
	GS-CR3	GGGUAAUUUCUACUAAGUGUAGAUCUCCUCUUAUUACUUACAUGGUU
ssDNA reporter	QF	FAM-TTATT-BHQ1
	LF	FAM-TTTTTTTATTTTTTT-Biotin
TaqMan qPCR	GSQ-F	TCCAGTCTATTCGGGTAGGAA
	GSQ-R	GACACCATGAAGCTCCACTAA
	GS-Q-PROBE	FAM-AAAGCTTTGGTAGGTTCATCAGTTCCT-BHQ1

**Table 2:** Intra-assay and inter-assay reproducibility of the *L. garvieae* TaqMan qPCR.

Concentration of Standard Plasmid (Copies/Reaction)	Intra-Assay Mean ± SD	Intra-Assay CV (%)	Inter-Assay Mean ± SD	Inter-Assay CV (%)
10^9^	14.70 ± 0.05	0.34	14.73 ± 0.17	1.15
10^8^	17.36 ± 0.05	0.29	17.28 ± 0.13	0.75
10^7^	20.63 ± 0.07	0.35	20.53 ± 0.14	0.68
10^6^	23.86 ± 0.09	0.36	23.80 ± 0.27	1.13
10^5^	27.05 ± 0.06	0.22	27.10 ± 0.19	0.70
10^4^	30.22 ± 0.13	0.43	30.16 ± 0.18	1.13
10^3^	33.43 ± 0.08	0.24	33.48 ± 0.20	0.60
10^2^	36.76 ± 0.14	0.38	36.85 ± 0.19	0.52
20	39.02 ± 0.22	0.56	39.38 ± 0.28	0.71

**Table 3:** Clinical sample test result.

Methods	Positive Sample	Negative Sample	Sample Count	Detection Rate
One-pot ERA-CRISPR/Cas12a-Fluorescence	32	104	136	23.5%
One-pot ERA-CRISPR/Cas12a-LFD	32	104	136	23.5%
TaqMan qPCR (this study)	32	104	136	23.5%
TaqMan qPCR [24]	32	104	136	23.5%

**Table 4:** Different molecular diagnostic methods for the detection for *L. garvieae*.

Methods	Target Gene	Accession No.	Templates	LOD	Time	References
PCR	16S rRNA	X54262	Bacterial suspension	36 CFU/mL	135.5 min	Zlotkin, et al. 1998 [21]
PCR	Dihydropteroate synthase gene	AB024530	/	/	77.5 min	Aoki et al. 2000 [58]
SYBR Green qPCR	16S rRNA	/	Genomic DNA	32 fg/μL	115 min	Jung, et al. 2010 [22]
SYBR Green qPCR	16S-23S rRNA ITS region	/	Genomic DNA	2.63 fg/μL	72.5 min	Thanh et al. 2012 [59]
LAMP	α/β fold family hydrolase gene	JF766366	Bacterial suspension	300 CFU/mL	60 min	Tsai, et al. 2013 [45]
Multiplex TaqMan qPCR	16S-23S rRNA ITS region		/	/	50 min	Chapela, et al.2018 [25]
TaqMan qPCR	16S–23S rRNA ITS region	HM241913.1	Plasmid	4 copies/μL	61.5 min	Shahin, et al. 2022 [60]
Multiplex PCR	DUF1430 domain-containing protein	AP009333.1	Bacterial suspension	5 CFU/mL	65 min	Ustaoglu, et al. 2024 [61]
Multiplex TaqMan qPCR	Hypothetical protein gene	SFL50150.1	Plasmid	9.73 copies/μL	60 min	Shahin, et al. 2025 [24]
One-pot ERA-CRISPR/Cas12a-Fluorescence	Hypothetical protein gene	AP009332.1	Plasmid	10 copies/reaction	30 min	This study
One-pot ERA-CRISPR/Cas12a-LFD				10 copies/reaction	35 min	This study
TaqMan qPCR				20 copies/reaction	55 min	This study

## Data Availability

The original contributions presented in this study are included in the article. Further inquiries can be directed to the corresponding author.

## Supplementary Materials

The following supporting information can be downloaded at: https://www.mdpi.com/article/10.3390/microorganisms14040830/s1, Supplementary Table S1: clinical isolates of *Lactococcus garvieae*. Supplementary Table S2: clinical examination samples.
