# Supplementary material for: Establishment of One-Pot ERA-CRISPR/Cas12a-Based Rapid Visual Assays and a TaqMan Quantitative PCR Assay for Lactococcus garvieae
Source: Microorganisms. 2026 Apr 5;14(4):830. doi: 10.3390/microorganisms14040830 (PMC13118430; doi:10.3390/microorganisms14040830)
Supplement: Supplementary file 1 [file microorganisms-14-00830-s001.zip › microorganisms-4200861-supplementary.pdf]

Supplementary materials

Table S1 Clinical isolates of *Lactococcus garvieae*.

Isolates ID	Country	Region	Isolation year	Host species	Isolation source
ATCC49156	Japan	Kochi	1974	*Seriola quinqueradiata*	Kidney
ATCC43921	Japan	Unknown	1973	Cow	Unknown
1	China	Guangdong	2020	*Oreochromis niloticus*	Kidney
2	China	Guangxi	2020	*Trachinotus* spp.	Spleen
3	China	Guangdong	2020	*Trachinotus* spp.	Kidney
4	China	Guangxi	2021	*Trachinotus* spp.	Kidney
5	China	Guangxi	2021	*Trachinotus* spp.	Liver
6	China	Guangxi	2021	*Trachinotus* spp.	Kidney
7	China	Guangdong	2021	*Siganus* spp.	Kidney
8	China	Guangdong	2021	*Siganus* spp.	Kidney
9	China	Guangdong	2021	*Terapon jarbua*	Kidney
10	China	Guangdong	2021	*Trachinotus* spp.	Kidney
11	China	Guangdong	2021	*Trachinotus* spp.	Kidney
12	China	Hainan	2021	*Trachinotus* spp.	Head kidney
13	China	Hainan	2021	*Trachinotus* spp.	Spleen
14	China	Hainan	2021	*Trachinotus*	Kidney

				spp.	
15	China	Guangxi	2021	*Trachinotus* spp.	Kidney
16	China	Hainan	2021	*Trachinotus* spp.	Spleen
17	China	Hainan	2021	*Trachinotus* spp.	Head kidney
18	China	Guangxi	2021	*Trachinotus* spp.	Kidney
19	China	Guangxi	2021	*Trachinotus* spp.	Liver
20	China	Guangxi	2021	*Trachinotus* spp.	Spleen
21	China	Guangdong	2021	*Rachycentron canadum*	Spleen
22	China	Guangdong	2021	*Rachycentron canadum*	Kidney
23	China	Guangdong	2021	*Trachinotus* spp.	Kidney
24	China	Guangdong	2021	*Trachinotus* spp.	Kidney
25	China	Guangdong	2021	*Trachinotus* spp.	Kidney
26	China	Guangxi	2021	*Trachinotus* spp.	Kidney
27	China	Guangxi	2021	*Trachinotus* spp.	Kidney
28	China	Guangdong	2021	*Trachinotus* spp.	Kidney
29	China	Guangdong	2022	*Trachinotus* spp.	Kidney
30	China	Guangdong	2022	*Trachinotus* spp.	Kidney

31	China	Guangdong	2022	*Trachinotus* spp.	Head kidney
32	China	Guangdong	2022	*Trachinotus* spp.	Kidney
33	China	Guangdong	2022	*Trachinotus* spp.	Kidney
34	China	Guangdong	2022	*Rachycentron canadum*	Kidney
35	China	Guangdong	2022	*Rachycentron canadum*	Kidney
36	China	Guangdong	2022	*Trachinotus* spp.	Kidney
37	China	Guangdong	2022	*Trachinotus* spp.	Kidney
38	China	Guangdong	2022	*Trachinotus* spp.	Spleen
39	China	Guangdong	2022	*Rachycentron canadum*	Kidney
40	China	Guangdong	2022	*Rachycentron canadum*	Head kidney
41	China	Guangdong	2023	*Rachycentron canadum*	Kidney
42	China	Guangdong	2023	*Rachycentron canadum*	Spleen
43	China	Guangdong	2023	*Trachinotus ovatus*	Kidney
44	China	Guangdong	2023	*Rachycentron canadum*	Kidney
45	China	Guangdong	2024	*Trachinotus* spp.	Kidney
46	China	Guangxi	2024	*Trachinotus* spp.	Kidney
47	China	Guangdong	2024	*Trachinotus*	Kidney

				spp.	
48	China	Guangdong	2024	*Rachycentron canadum*	Spleen
49	China	Guangdong	2024	*Rachycentron canadum*	Spleen
50	China	Guangdong	2024	*Seriola dumerili*	Kidney
51	China	Guangdong	2024	*Seriola dumerili*	Kidney
52	China	Guangdong	2024	*Rachycentron canadum*	Head kidney
53	China	Guangdong	2025	*Trachinotus* spp.	Kidney
54	China	Guangxi	2025	*Trachinotus* spp.	Kidney
55	China	Guangdong	2025	*Rachycentron canadum*	Kidney
56	China	Guangdong	2025	*Trachinotus* spp.	Kidney
57	China	Guangdong	2025	*Trachinotus* spp.	Head kidney

Table S2 Clinical examination samples.

Number	Country	Region	Host species	Detection results
1	China	Guangdong	*Trachinotus* spp.	Positive
2	China	Guangdong	*Trachinotus* spp.	Positive
3	China	Guangdong	*Trachinotus* spp.	Negative
4	China	Guangdong	*Trachinotus* spp.	Negative

5	China	Guangdong	*Trachinotus* spp.	Positive
6	China	Guangdong	*Trachinotus* spp.	Positive
7	China	Guangdong	*Trachinotus* spp.	Negative
8	China	Guangdong	*Trachinotus* spp.	Positive
9	China	Guangdong	*Trachinotus* spp.	Positive
10	China	Guangdong	*Trachinotus* spp.	Negative
11	China	Guangdong	*Trachinotus* spp.	Negative
12	China	Guangdong	*Trachinotus* spp.	Positive
13	China	Guangdong	*Trachinotus* spp.	Negative
14	China	Guangdong	*Trachinotus* spp.	Negative
15	China	Guangdong	*Trachinotus* spp.	Negative
16	China	Guangdong	*Trachinotus* spp.	Positive
17	China	Guangdong	*Trachinotus* spp.	Negative
18	China	Guangdong	*Trachinotus* spp.	Negative
19	China	Guangdong	*Trachinotus* spp.	Positive
20	China	Guangdong	*Trachinotus* spp.	Negative
21	China	Guangdong	*Trachinotus*	Negative

			spp.	
22	China	Guangdong	*Trachinotus* spp.	Positive
23	China	Guangdong	*Trachinotus* spp.	Negative
24	China	Guangdong	*Trachinotus* spp.	Positive
25	China	Guangdong	*Trachinotus* spp.	Positive
26	China	Guangdong	*Trachinotus* spp.	Negative
27	China	Guangdong	*Trachinotus* spp.	Positive
28	China	Guangdong	*Trachinotus* spp.	Positive
29	China	Guangxi	*Trachinotus* spp.	Negative
30	China	Guangxi	*Trachinotus* spp.	Negative
31	China	Guangxi	*Trachinotus* spp.	Negative
32	China	Guangxi	*Trachinotus* spp.	Negative
33	China	Guangxi	*Trachinotus* spp.	Positive
34	China	Guangxi	*Trachinotus* spp.	Negative
35	China	Guangxi	*Trachinotus* spp.	Positive
36	China	Guangxi	*Trachinotus* spp.	Negative
37	China	Guangdong	*Rachycentron canadum*	Negative

38	China	Guangdong	*Rachycentron canadum*	Negative
39	China	Guangdong	*Rachycentron canadum*	Negative
40	China	Guangdong	*Rachycentron canadum*	Positive
41	China	Guangdong	*Rachycentron canadum*	Negative
42	China	Guangdong	*Rachycentron canadum*	Positive
43	China	Guangdong	*Rachycentron canadum*	Negative
44	China	Guangdong	*Rachycentron canadum*	Positive
45	China	Guangdong	*Rachycentron canadum*	Positive
46	China	Guangdong	*Rachycentron canadum*	Negative
47	China	Guangdong	*Rachycentron canadum*	Negative
48	China	Guangdong	*Rachycentron canadum*	Positive
49	China	Guangdong	*Rachycentron canadum*	Positive
50	China	Guangdong	*Rachycentron canadum*	Negative
51	China	Guangdong	*Rachycentron canadum*	Negative
52	China	Guangdong	*Rachycentron canadum*	Negative
53	China	Guangdong	*Rachycentron canadum*	Positive
54	China	Guangdong	*Rachycentron*	Negative

			canadum	
55	China	Guangdong	*Rachycentron canadum*	Negative
56	China	Guangdong	*Rachycentron canadum*	Negative
57	China	Guangdong	*Rachycentron canadum*	Positive
58	China	Guangdong	*Rachycentron canadum*	Negative
59	China	Guangdong	*Rachycentron canadum*	Positive
60	China	Guangdong	*Rachycentron canadum*	Positive
61	China	Guangdong	*Rachycentron canadum*	Positive
62	China	Guangdong	*Rachycentron canadum*	Negative
63	China	Guangdong	*Rachycentron canadum*	Negative
64	China	Guangdong	*Rachycentron canadum*	Positive
65	China	Guangdong	*Rachycentron canadum*	Negative
66	China	Guangdong	*Rachycentron canadum*	Negative
67	China	Guangdong	*Rachycentron canadum*	Negative
68	China	Guangdong	*Rachycentron canadum*	Negative
69	China	Guangdong	*Rachycentron canadum*	Negative
70	China	Guangdong	*Rachycentron canadum*	Negative

71	China	Guangdong	*Plectropomus leopardus*	Positive
72	China	Guangdong	*Plectropomus leopardus*	Negative
73	China	Guangdong	*Plectropomus leopardus*	Negative
74	China	Guangdong	*Plectropomus leopardus*	Negative
75	China	Guangdong	*Plectropomus leopardus*	Negative
76	China	Guangdong	*Plectropomus leopardus*	Negative
77	China	Guangdong	*Plectropomus leopardus*	Negative
78	China	Guangdong	*Plectropomus leopardus*	Negative
79	China	Hainan	*Plectropomus leopardus*	Negative
80	China	Hainan	*Plectropomus leopardus*	Negative
81	China	Hainan	*Plectropomus leopardus*	Negative
82	China	Hainan	*Plectropomus leopardus*	Negative
83	China	Hainan	*Plectropomus leopardus*	Negative
84	China	Hainan	*Plectropomus leopardus*	Negative
85	China	Hainan	*Plectropomus leopardus*	Negative
86	China	Hainan	*Plectropomus leopardus*	Negative
87	China	Hainan	*Plectropomus*	Negative

			leopardus	
88	China	Hainan	*Plectropomus leopardus*	Negative
89	China	Hainan	*Plectropomus leopardus*	Negative
90	China	Hainan	*Plectropomus leopardus*	Negative
91	China	Hainan	*Plectropomus leopardus*	Negative
92	China	Guangdong	pearl gentian grouper	Negative
93	China	Guangdong	pearl gentian grouper	Negative
94	China	Guangdong	pearl gentian grouper	Negative
95	China	Guangdong	pearl gentian grouper	Negative
96	China	Guangdong	pearl gentian grouper	Negative
97	China	Guangdong	pearl gentian grouper	Negative
98	China	Guangdong	pearl gentian grouper	Negative
99	China	Guangdong	pearl gentian grouper	Positive
100	China	Guangdong	pearl gentian grouper	Negative
101	China	Guangdong	pearl gentian grouper	Negative
102	China	Guangxi	pearl gentian grouper	Negative
103	China	Guangxi	pearl gentian grouper	Negative

104	China	Guangxi	pearl gentian grouper	Negative
105	China	Guangxi	pearl gentian grouper	Negative
106	China	Hainan	pearl gentian grouper	Negative
107	China	Guangdong	*Seriola dumerili*	Negative
108	China	Guangdong	*Seriola dumerili*	Negative
109	China	Guangdong	*Seriola dumerili*	Negative
110	China	Guangdong	*Seriola dumerili*	Positive
111	China	Guangdong	*Seriola dumerili*	Negative
112	China	Guangdong	*Seriola dumerili*	Negative
113	China	Guangdong	*Seriola dumerili*	Negative
114	China	Guangdong	*Seriola dumerili*	Positive
115	China	Guangdong	*Seriola dumerili*	Negative
116	China	Guangdong	*Seriola dumerili*	Negative
117	China	Guangdong	*Seriola dumerili*	Negative
118	China	Guangdong	*Seriola dumerili*	Negative
119	China	Guangdong	*Siganus* spp.	Negative
120	China	Guangdong	*Siganus* spp.	Negative
121	China	Guangdong	*Siganus* spp.	Negative

122	China	Guangdong	*Siganus* spp.	Negative
123	China	Guangdong	*Siganus* spp.	Negative
124	China	Guangdong	*Siganus* spp.	Negative
125	China	Guangxi	*Siganus* spp.	Negative
126	China	Guangxi	*Siganus* spp.	Negative
127	China	Guangdong	*Thamnaconus modestus*	Negative
128	China	Guangdong	*Thamnaconus modestus*	Negative
129	China	Guangdong	*Thamnaconus modestus*	Negative
130	China	Guangdong	*Thamnaconus modestus*	Negative
131	China	Guangdong	*Thamnaconus modestus*	Negative
132	China	Guangdong	*Thamnaconus modestus*	Negative
133	China	Guangdong	*Thamnaconus modestus*	Negative
134	China	Guangdong	*Selenotoca multifasciata*	Negative
135	China	Guangdong	*Selenotoca multifasciata*	Negative
136	China	Guangdong	*Selenotoca multifasciata*	Negative
